# Loss of Acid Sensing Ion Channel-1a and Bicarbonate Administration Attenuate the Severity of Traumatic Brain Injury

**DOI:** 10.1371/journal.pone.0072379

**Published:** 2013-08-26

**Authors:** Terry Yin, Timothy E. Lindley, Gregory W. Albert, Raheel Ahmed, Peter B. Schmeiser, M. Sean Grady, Matthew A. Howard, Michael J. Welsh

**Affiliations:** 1 Department of Internal Medicine, Roy J. and Lucille A. Carver College of Medicine, University of Iowa, Iowa City, Iowa, United States of America; 2 Department of Neurosurgery, Roy J. and Lucille A. Carver College of Medicine, University of Iowa, Iowa City, Iowa, United States of America; 3 Howard Hughes Medical Institute, Roy J. and Lucille A. Carver College of Medicine, University of Iowa, Iowa City, Iowa, United States of America; 4 Department of Neurosurgery, Perelman School of Medicine, University of Pennsylvania, Philadelphia, Pennsylvania, United States of America; The Ohio State University, United States of America

## Abstract

Traumatic brain injury (TBI) is a common cause of morbidity and mortality in people of all ages. Following the acute mechanical insult, TBI evolves over the ensuing minutes and days. Understanding the secondary factors that contribute to TBI might suggest therapeutic strategies to reduce the long-term consequences of brain trauma. To assess secondary factors that contribute to TBI, we studied a lateral fluid percussion injury (FPI) model in mice. Following FPI, the brain cortex became acidic, consistent with data from humans following brain trauma. Administering HCO_3_^−^ after FPI prevented the acidosis and reduced the extent of neurodegeneration. Because acidosis can activate acid sensing ion channels (ASICs), we also studied *ASIC1a^−/−^* mice and found reduced neurodegeneration after FPI. Both HCO_3_^−^ administration and loss of ASIC1a also reduced functional deficits caused by FPI. These results suggest that FPI induces cerebral acidosis that activates ASIC channels and contributes to secondary injury in TBI. They also suggest a therapeutic strategy to attenuate the adverse consequences of TBI.

## Introduction

Traumatic brain injury (TBI) is a common public health issue that affects an estimated 1.7 million individuals annually in the United States [1]. The pathophysiology of TBI involves at least two stages. The initial mechanical event causes structural damage to brain tissue. That primary injury is followed by a secondary injury, a complex process that can extend from minutes to months and leads to impaired function and death of neurons [2]–[4]. The acute and typically unpredictable timing of the primary injury supports the adoption of preventive measures, but also accounts for the many practical problems and constraints associated with immediate therapeutic intervention [5]. However, the more prolonged time during which secondary injury progresses suggests that there is a window of opportunity to intervene and reduce the adverse consequences and long-term sequelae of TBI.

Multiple potential mechanisms for secondary TBI have been identified and include reduced cerebral blood flow causing brain ischemia [6]–[8], brain edema causing increased intracranial pressure that can further impair cerebral blood flow and cause herniation syndromes [9], [10], excitotoxicity [11]–[13], oxidative stress [14]–[16], and inflammation [17]–[20]. Acidosis is also a factor that could contribute to the secondary injury. Previous studies have shown that brain tissue pH falls after TBI, and the reduction is greater in patients with more severe injury [21]–[25]. Acidosis may be due to ischemia and to metabolic changes induced by the injury [26].

A reduced pH could potentially enhance TBI severity by activating acid sensing ion channels (ASICs) [27]–[31]. ASICs are members of the degenerin/epithelial Na^+^ channel family of ion channels and are widely expressed in the central nervous system [32]–[35]. Neuronal ASIC channels are hetero-trimers [36] composed primarily of ASIC1a and ASIC2 subunits [32]–[35]. These subunits form non-voltage gated channels that are activated by a reduction of extracellular pH and conduct Na^+^ and to a lesser extent Ca^2+^. The ASIC1a subunit is required for currents in response to acidic stimuli of less than pH 5 as demonstrated in neurons from mice with a disrupted ASIC1a gene (*ASIC1a^−/−^* mice) [35], [37].

Previous studies have shown that acidosis can cause neuronal injury that is at least in part mediated by ASIC channels. For example, extracellular acidosis accompanying cerebral ischemic stroke contributed to neuronal injury, and eliminating ASIC1a from neurons attenuated acidosis-induced cell injury and death [38]–[40]. Inhibiting ASICs also attenuated neuronal injury following reperfusion after an ischemic insult in rats [40]. Extracellular pH also falls during autoimmune encephalomyelitis, a mouse model of multiple sclerosis, and loss of ASIC1a in *ASIC1a^−/−^* mice was neuroprotective [41]. It has also been proposed that ASIC channels contribute to the pathological consequences of Parkinson’s disease [42]–[44] and Huntington’s disease [45]. Earlier studies of TBI in rats examined the effect of amiloride, which can inhibit ASIC currents. One study suggested that amiloride administered before TBI could attenuate severity [46], and another study suggested that amiloride administered 30 min after TBI exacebated the injury [47]. Further limiting the interpretation of such studies, amiloride can also inhibit the type-1 Na^+^-H^+^ exchanger and the Na^+^-Ca^2+^ exchanger.

Based on this background, we hypothesized that TBI induces an acidosis that activates ASIC channels and thereby contributes to the neuronal injury following mechanical trauma. To test this hypothesis, we used the lateral fluid percussion injury (FPI) model in which a fluid pressure pulse is delivered to the intact dura [48]. The lateral FPI model is one of the most widely used animal models of TBI because it provides an injury of reproducible severity [49].

## Results

### FPI Causes Cerebral Acidosis that is Prevented by HCO_3_^−^ Administration

**Figure 1:**
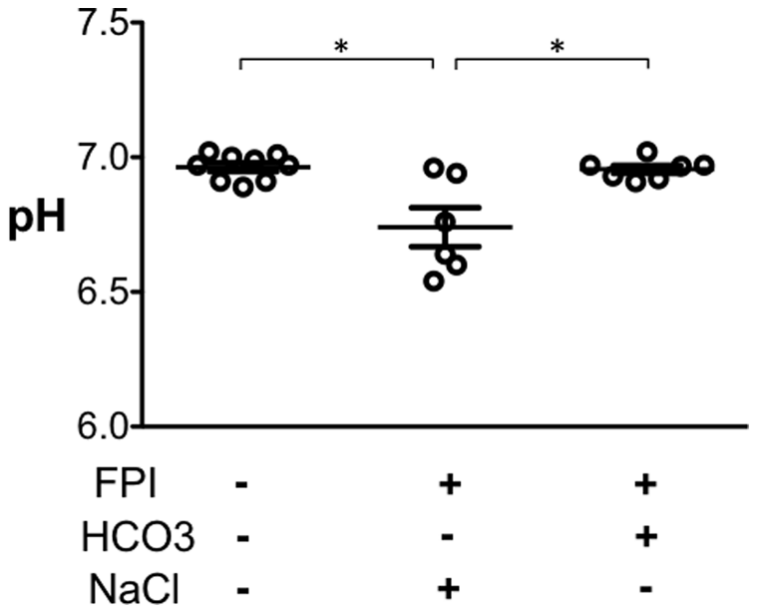
HCO_3_^−^ administration prevents the FPI-induced decrease in brain pH. Data are cortical brain pH one hr after FPI. NaHCO_3_ was administered as indicated immediately after FPI. Animals were anesthetized and mechanically ventilated. N = 9 control, 6 FPI, and 7 FPI plus NaHCO_3_ mice. Each data point indicates an individual animal. * indicates P<0.05; ANOVA with Bonferroni post-hoc test.

Based on reports that TBI reduces brain pH in humans [21], [22] and rats [50], we tested the hypothesis that FPI induces cerebral acidosis in mice. We prepared mice with a craniotomy and placed a modified plastic hub on the right parietal bone. One day later mice were anesthetized and one group received an FPI and the other was sham-treated. We then inserted a fiber-optic pH probe through the craniotomy site into parietal cortex and measured pH one hour after FPI. Because TBI and anesthesia might change ventilation and thereby pH, we ventilated animals to maintain a constant PCO_2_. Compared to the sham-treated mice, pH was reduced in mice that received FPI (Fig. 1).

An earlier study showed that HCO_3_^−^ administration raised the pH of brain tissue and cerebral spinal fluid [51]. Moreover, delivering HCO_3_^−^ blunted the acidosis induced by breathing CO_2_ [51]. We found that delivering intraperitoneal NaHCO_3_ immediately after FPI prevented the FPI-induced reduction in pH (Fig. 1). These results indicate that as in humans with TBI, FPI produced cerebral acidosis in mice. Moreover, administering HCO_3_^−^ prevented the pH drop.

### Eliminating ASIC1a or Administering HCO_3_^−^ Attenuates FPI-induced Neurodegeneration

**Figure 2:**
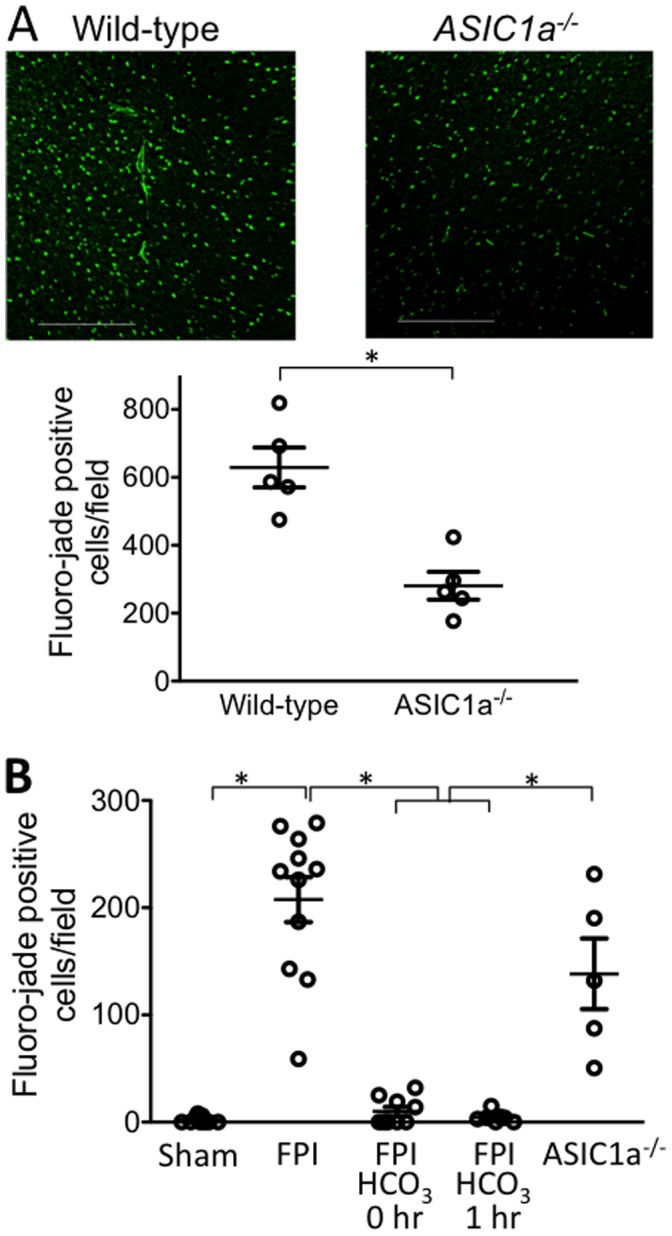
Loss of ASIC1a and NaHCO_3_ administration reduce FPI-induced neuronal degeneration. **A.** Brain was removed one day after FPI. Data are fluoro-jade positive neurons per microscopic field in the cortex (0.9 μm^2^). N = 5 wild-type and 5 *ASIC1a^−/−^* mice. Scale bar = 0.1 mm. Each data point indicates an individual animal. * indicates P<0.05, unpaired t-test. **B.** Brain was removed four days after FPI. N = 9 sham-treated mice, 11 FPI-treated wild-type mice 3 of which received NaCl after FPI, 9 FPI-treated mice with NaHCO_3_ administered immediately after FPI, 7 FPI-treated mice with NaHCO_3_ administered 1 hr after FPI, and 5 FPI-treated *ASIC1a^−/−^* mice. * indicates P<0.05, ANOVA with Bonferroni post-hoc test.

Previous work has shown that FPI causes the death of neurons [52], [53]. We asked if disrupting the *ASIC1a* gene, and thereby eliminating ASIC-dependent acid-evoked currents, would attenuate neuronal degeneration. We used fluoro-jade staining to detect degenerating neurons [54], [55]. Observers unaware of the treatment condition counted fluoro-jade-stained neurons in standardized parts of the ipsilateral cortex. One day after FPI, we found abundant staining in wild-type mice (Fig. 2A). In contrast, *ASIC1a^−/−^* mice had approximately half as many fluoro-jade positive cells. By four days after the injury, fewer fluoro-jade positive cells were observed in both the wild-type and *ASIC^−/−^* mice (Fig. 2B), which likely reflects death of cells that were previously labeled. The reduction in fluoro-jade stained cells in the *ASIC^−/−^* mice was not statistically different from that in the wild-type mice.

The FPI-induced fall in pH and attenuation of neuronal degeneration by eliminating ASIC1a channels suggested that an acidic pH was responsible. To test that hypothesis, we administered HCO_3_^−^ immediately after the FPI. Compared to control mice, animals that received HCO_3_^−^ had a reduction in fluoro-jade staining (Fig. 2B). We also tested the effect of delivering the HCO_3_^−^ 1 hr after the FPI. As with immediate administration, we observed a substantial reduction in the number of fluoro-jade positive cells. These results suggest that a reduced pH activates ASIC channels and exacerbates neuronal degeneration. However, the greater reduction in neurodegeneration with HCO_3_^−^ administration compared to *ASIC1a* gene disruption suggests that the reduced pH had additional injurious effects.

### Loss of ASIC1a and HCO_3_^−^ Administration Prevent FPI-induced Deficits in Spatial Memory

**Figure 3:**
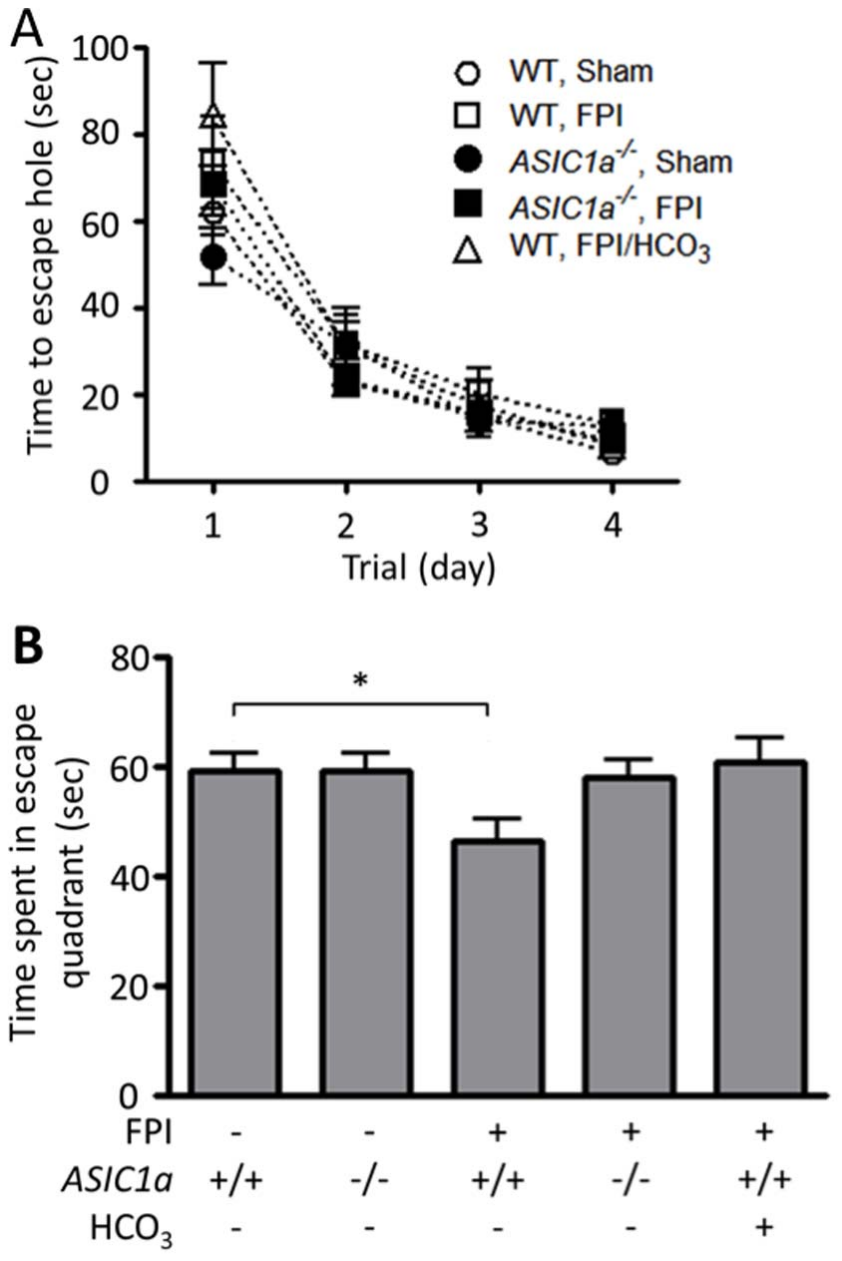
Disruption of the *ASIC1a* gene and NaHCO_3_ administration reduce the FPI-induced decrement in spatial memory. **A.** Data from 4 days of training on the Barnes maze. Data are latency to find the escape hole. N = 11 wild-type, sham-treated mice, 17 FPI-treated wild-type mice, 14 *ASIC1a^−/−^* sham-treated mice, 19 *ASIC1a^−/−^* FPI-treated mice, and 10 FPI-treated wild-type mice that received NaHCO_3_ immediately after FPI. None of the groups showed a statistically significant difference. **B.** Data are time during 90 sec. that mice spent in the quadrant that had contained the escape hole. * indicates P<0.05 compared to sham treated wild-type mice; ANOVA with Bonferroni post-hoc test.

FPI can impair hippocampal function and hippocampus-dependent spatial memory [56], [57]. Our finding that disrupting the *ASIC1a* gene and HCO_3_^−^ treatment protected neurons from FPI suggested that these interventions would also attenuate spatial memory deficits. To test this hypothesis, we used a Barnes maze to assess hippocampus-dependent spatial learning and memory [58]; previous work showed that TBI impairs performance on a Barnes maze [59], [60]. In the Barnes maze, mice must use spatial memory to locate an escape hole among 43 holes around the circumference of a circular table. Mice were studied on four consecutive days, during which time we measured the latency to reach the escape hole (Fig. 3A). During this acquisition phase of the assay, neither FPI, loss of ASIC1a nor HCO_3_^−^ administration had a statistically significant effect on latency to find the escape hole.

On the fifth day, we performed a probe trial to test for memory of the escape hole (Fig. 3B). The escape nest was removed, and we measured the amount of time mice spent in the quadrant that had contained the escape hole. Sham-treated wild-type and *ASIC1a^−/−^* mice spent a similar amount of time in the escape quadrant. In wild-type mice, FPI reduced the time in the escape quadrant. In contrast, FPI had no effect on performance of the *ASIC1a^−/−^* mice. Treating wild-type mice with HCO_3_^−^ after FPI also prevented a reduction in time spent in the escape quadrant. These results are consistent with our studies of neuronal degeneration and suggest that eliminating ASIC1a or preventing a reduction in brain pH can at least partially attenuate the adverse effects of TBI on spatial learning.

### HCO_3_^−^ Administration Restores Contextual Fear Behavior After FPI

**Figure 4:**
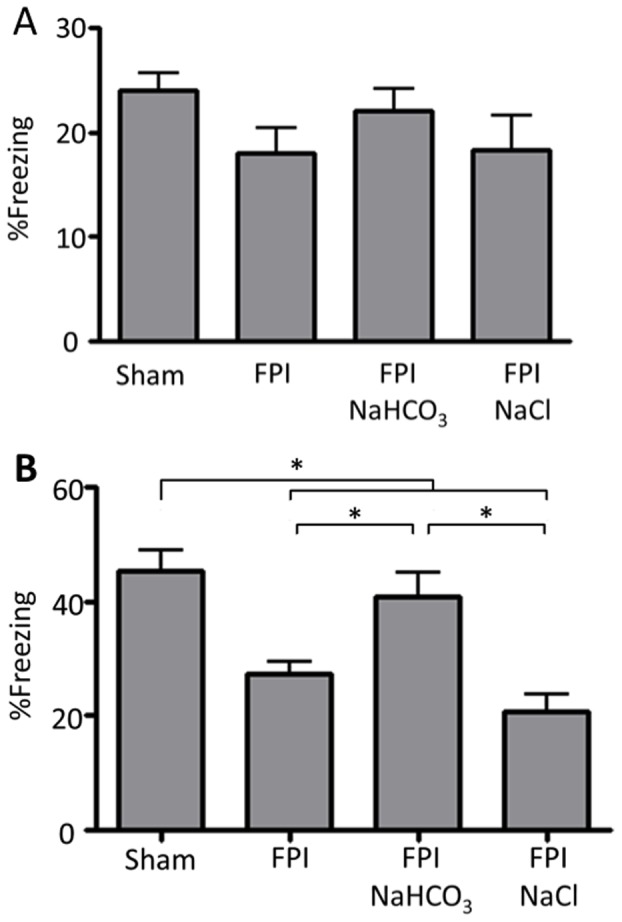
NaHCO_3_ administration improves performance in fear conditioning assay done after FPI. **A.** Percentage of time freezing during 8 min on the training day. All the mice were wild-type. N = 25 sham-treated, 10 FPI-treated controls, 14 FPI-treated with NaHCO_3_ administered immediately after FPI, and 6 FPI-treated with NaCl administered immediately after FPI. There were no statistically significant differences between groups. **B.** Percentage of time freezing during 6 min on the testing day. * indicates P<0.05; ANOVA with Bonferroni post-hoc test. ** indicates P<0.05 compared to FPI control and FPI treated with NaCl; ANOVA with Bonferroni post-hoc test.

ASIC1a is more abundantly expressed in the amygdala than in the hippocampus [37]. Contextual fear conditioning assays depend on amygdala function, in addition to hippocampal function [61]–[63]. Moreover, our earlier work showed that loss of ASIC1a decreased contextual fear conditioning [37]; because of that performance defect, we did not test them with FPI. In the fear conditioning assay, mice are trained to associate a novel environment, a context, with a foot shock, and freezing behavior is measured as an expression of a threat-induced defense reaction. During the training phase of fear conditioning, mice received five foot shocks, and we recorded the percentage of time they spent freezing (Fig. 4A). FPI did not significantly alter freezing behavior.

Twenty-four hours after training, we returned the mice to the same context and measured the percentage of time they spent freezing in the absence of foot shocks (Fig. 4B). Compared to sham-treated animals, FPI reduced the threat-induced defense reaction by ∼40%. However, mice that had received a NaHCO_3_^−^ injection following FPI showed less freezing behavior, and the time they spent freezing did not differ from that of sham-treated animals. In contrast, mice that received NaCl instead of NaHCO_3_ showed no improvement in freezing behavior.

## Discussion

Our data indicate that FPI induced brain acidosis that contributed to neuronal injury through ASIC channels. We discovered that reducing brain acidosis by administering HCO_3_^−^ decreased neuronal degeneration and improved performance in tests that assess spatial memory and memory involving the reaction to threat. Likewise, eliminating a target of an acidic pH, ASIC1a channels, reduced neuronal degeneration and improved performance in spatial learning. In addition, we found that HCO_3_^−^ administration was more effective than *ASIC1a* gene disruption at preventing neurodegeneration. That result suggests that the acidic brain pH may have had injurious effects in addition to activation of ASIC channels.

The results of these studies suggest that cerebral acidosis plays an important role in the secondary injury that is a key component of TBI. Our findings have similarities to observations that ischemic stroke and a model of multiple sclerosis induce cerebral acidosis and that their severity is reduced by disrupting the *ASIC1a* gene [38], [39], [41]. Reports that ASIC channels may play a role in reperfusion injury [40], Huntington’s disease [45], and Parkinson’s disease [42]–[44] suggest that in those disorders cerebral acidosis might also contribute to disease severity. In these various diseases, the mechanisms that produce acidosis could differ, but acidosis may be a common pathway leading to neuronal pathology. Although our data suggest that a reduced pH may contribute to injury via more than one mechanism, activation of ASIC channels may be one key component. ASIC channels could contribute to an increased [Ca^2+^]_i_ leading to cell injury and death by several mechanisms [64]. ASICs have a small Ca^2+^ conductance that could contribute, and Na^+^ entry could depolarize the cell membrane thereby activating voltage-gated Ca^2+^ channels. Depolarization might induce excitotoxicity. Activation of ASIC channels depends on their subunit composition [65]. In cultured neurons, pH values of 6.9–7.2 can activate these currents, suggesting that they can respond to small pH changes [32], [51], [66]–[68]. These considerations also lead us to speculate that acidosis and activation of ASICs might contribute to other diseases involving neuron injury and degeneration.

Our study has certain strengths as well as a number of limitations. An advantage of the experimental design is that we measured the effect of the interventions – HCO_3_^−^ administration and *ASIC1a* gene disruption – using both structural and functional assays and obtained concordant results. Use of *ASIC1a^−/−^* mice also has an advantage compared to inhibiting ASIC currents with pharmacological agents, which can often have partial effects on the intended target and introduce additional unanticipated effects. A limitation of our work is the use of mice and the FPI model; there are several species and models of TBI, and each has advantages and disadvantages [49]. Thus, it may be of value to extend this work to other models. Our assay of brain pH probably does not provide an accurate measure of absolute extracellular pH because inserting the pH probe will cause local injury, and CNS depression and mechanical ventilation might also alter pH. Nevertheless, the probe likely indicates changes in pH based on our study and previous work [51], [69] showing that the probe reports expected changes in pH with CO_2_ inhalation and HCO_3_^−^ administration. In addition, similar pH electrode methods have been used in other studies [70], [71]. Our measurements indicate that extracellular brain pH fell after FPI, but we were unable to determine whether intracellular pH changed, and a reduced intracellular pH might also contribute to injury. Moreover, a decreased intracellular pH could affect many processes other than ASIC channels. To carry this work toward therapeutics, it would also be valuable to measure blood gases and serum HCO_3_^−^ concentrations.

In this study, we tested one aspect of the secondary injury component of TBI. However, several additional factors may contribute to secondary aspects of TBI, and our data do not exclude other proposed mechanisms. For example, other processes involved in TBI include brain ischemia [6]–[8], brain edema causing increased intracranial pressure [9], [10], excitotoxicity [11]–[13], oxidative stress [14]–[16], and inflammation [17]–[20]. In addition, several potential treatments have shown some promise in TBI including anti-depressants [61], [62] and progesterone [72], [73]. These studies emphasize the complexity of TBI and suggest that understanding the pathophysiological mechanisms is essential in order to rationally develop new therapies.

A challenge in managing TBI is to develop neuroprotective strategies that will reduce the secondary injury that follows the initial mechanical insult. Previous studies have focused on brain acidosis as a potential therapeutic target in management of head injury. CSF pH is reduced in humans during the acute phase after head injury and greater reductions in pH are associated with more severe injury and worse clinical outcomes [21]–[25]. Our experiments suggest that minimizing brain acidosis and/or inhibiting ASIC channels might have the potential to interfere with that pathway and thereby attenuate functional deficits. Two observations suggest the potential feasibility of pursuing such a strategy. First, TBI causes a reduction in brain pH in humans [21], [22]. Second, it may be possible to alter brain pH in humans. Although brain pH was not measured, HCO_3_^−^ has been administered to patients with severe TBI [74], [75]; hypertonic NaHCO_3_ was given intravenously in place of hypertonic NaCl to reduce elevated intracranial pressures. While NaHCO_3_ was as effective as saline at reducing intracranial pressure, brain pH was not measured and whether HCO_3_^−^ administration had any additional long-term benefits remains uncertain. Patients with TBI are sometimes hyperventilated with a goal of managing intracranial pressure, and that maneuver can also elevate brain pH. However, in severe head injury, hyperventilated patients had a less favorable outcome, perhaps due to the associated adverse effects of hyperventilation [76]. pH buffers such as tormethamine have been tested in animals [77]. Despite initial promise, these therapeutic approaches have shown limited success for long term neurological outcomes in humans [78]–[80]. Thus, greater understanding of the underlying molecular mechanisms of acidosis-mediated injury and additional approaches may be beneficial in improving long-term neurological outcomes. Although our data indicate that administering NaHCO_3_ one hour after FPI minimizes brain injury in mice, a practical therapeutic strategy directed at reversing brain acidosis or inhibiting ASIC channels in humans has yet to be developed and tested. Nevertheless, the data suggest that normalizing brain pH and/or inhibiting ASIC might be effective in reducing functional deficits in patients who suffer a TBI.

## Methods

### Mice

#### Ethic statement

All animal protocols were approved by the University of Iowa Animal Care and Use Committee. We used male 2–4 month old wild-type and *ASIC1a^−/−^* mice on a congenic C57BL/6J background as previously described [35]. Littermates were used as controls. Mice were adapted to handling prior to testing in behavioral assays.

### Fluid Percussion Injury

We used methods similar to those previously described [48]. Briefly, animals were anesthetized with pentobarbital. Hair was removed from the skull, and mice were secured in a stereotactic holder. The skull was exposed, a 3 mm diameter hole was placed through the skull leaving the dura intact, and a modified Leur-lock hub was placed over the hole and secured with glue. The hub was filled with saline and capped. One day later, mice were anesthetized with 2% isoflurane. The Leur lock adapter was then connected to a fluid percussion injury device (Custom Design and Fabrication, Virginia Commonwealth University), which delivered a 0.9 atmosphere pulse. Following FPI, the Leur lock adapter was removed, the scalp sutured, and mice were monitored until fully recovered, at which time they were returned to their home cage.

### Fluoro-jade Staining

Mice were euthanized with pentobarbital, perfused with saline to clear blood, and then perfused with 4% paraformaldehyde. Brains were removed and soaked in 4% paraformaldehyde overnight at 4°C. Brains were then immersed in a 30% sucrose solution until they were no longer buoyant and then frozen with liquid nitrogen. Ten μm thick sections of brain were cut at −28°C with a cryostat (HM 505E, Leica Microsystems, Wetzlar, Germany). Sections were mounted on Superfrost Plus slides (Fisher Scientific, Pittsburgh, PA). Slides were stained with fluoro-jade according to manufacturer’s directions (Millipore, Billerica, MA). Briefly, slides were air dried at 50°C for 25 min, placed in 1% NaOH in EtOH for 5 min, submerged in 70% EtOH for 2 min, washed in double distilled H_2_O for 2 min, immersed in 0.06% KMnO_4_ for 10 min, and washed again. Slides were then incubated in a solution of 50 μl acetic acid, 2 ml fluoro-jade and 50 ml double distilled H_2_O for 30 min. They were then washed 3 times, dried at 40°C for 15 min, submerged in 95% EtOH, 100% EtOH, and Xylems each for 1 min, air-dried and a cover slip was applied. They were imaged with an Olympus FluoView1000 confocal microscope (Center Valley, PA). Sections for examination were chosen from coronal sections of the cortex that were referenced to the anterior, medial, and posterior hippocampus. For each animal, we counted 3 microscopic fields of cortex from each of the 3 areas and averaged the data from 9 fields to generate the number of fluoro-jade positive cells per field for an individual animal. Operators were unaware of genotype or treatment.

### pH Measurement

Following FPI, a tracheostomy was placed and a mice were ventilated at 50 breaths/min, tidal volume 35 ml/Kg, and an I:E ratio of 1 with a rodent ventilator (SAR 830/P, CWE incorporated, Veemarktade, Netherlands). During ventilation, sedation was maintained with 2% isoflurane inhalation, and mice were paralyzed with pancronium (0.1 ml/kg, intraperitoneal). We monitored mice with electrocardiogram (emka Technologies, Falls Curch, VA). Cortical pH was measured with a fiber optic pH electrode (tip size 140 μm) (#502123, World Precision Instruments, Sarasota, FL). Probes were calibrated before and after each experiment with standard solutions ranging from pH 6 to 8 as described by the manufacturer. Probes were inserted 500 μm into the cortex at the site of craniotomy, and pH was recorded one hr after the FPI.

### Administration of NaHCO_3_ and NaCl

Some mice received an intraperitoneal injection of NaHCO_3_ or NaCl immediately or 1 hr after FPI. Animals received 2×10^−3^ moles/Kg of NaHCO_3_ or NaCl from 200 mM stock solutions; for example, a 25 g mouse received 250 μl of solution.

### Barnes Maze Assay

A Barnes maze assay was used to assess spatial memory. The protocol was similar to that previously described [58]. The apparatus was a circular table with a diameter of 122 cm, placed 95 cm above the ground. Forty four 5.1 cm diameter holes were located equidistantly around the perimeter. During the training period, an escape box containing bedding was placed under the escape hole. The position of the escape hole was varied for each mouse, but was constant during the training period. Each mouse was tested 4 times per day for 4 days; 15 min separated each trial. At the start of a trial, a mouse was placed on the center of the maze. The trial lasted 3 min or until the mouse found the escape hole. If at the end of 3 min the mouse had not found the escape hole, it was shown the location. Once a mouse entered the escape box it was allowed to remain there for 3–5 min. We measured the time (latency) that a mouse took to find and enter the escape hole. On the fifth day each mouse underwent a probe trial, during which the escape box was removed. The mouse was place in the center of the maze, and its position tracked for 90 sec. We recorded the percentage of time it spent in the quadrant that had contained the escape hole. During the training period and probe trial, movement of mice was recorded by video and scored later. Operators were not aware of genotype or treatment.

### Context Fear Conditioning Assay

We used methods for context fear conditioning similar to those previously described [63]. Experiments were performed using a computerized video fear conditioning system (Med Associates, St Albuns, VT). On day 1, the training day, mice were placed in a conditioning chamber for 4 min. They then received a 0.75 mAmp foot shock for 1 sec. A total of 5 shocks were delivered with 80 sec. intervals between shocks. Freezing behavior was measured during the 8 min training period. Freezing behavior was defined as the absence of movement other than respiration and was scored with VideoFreeze software (Med Associates). Mice were then removed and returned to their home cage. Twenty-four hours later, the testing day, mice were placed in the training chamber. No foot shocks were delivered, and freezing behavior was assessed for 5 min. Operators were not aware of genotype or treatment.

### Statistical Analysis

Statistical significance was evaluated using unpaired t-test. For multiple comparisons, we used an ANOVA with a Bonferroni post-hoc test. Differences were considered statistically significant with P<0.05.

## References

[1] Faul M, Xu L, Wald MD, Coronado VG (2010) Traumatic brain injury in the United States: emergency department visits, hospitalizations, and deaths. In: Centers for Disease Control and Prevention NCfIPaC, editor. Atlanta, GA.

[2] Blennow K, Hardy J, Zetterberg H (2012) The neuropathology and neurobiology of traumatic brain injury. Neuron 76: 886–899. 23217738 10.1016/j.neuron.2012.11.021

[3] Marion DW (1998) Head and spinal cord injury. Neurol Clin 16: 485–502. 9537971 10.1016/s0733-8619(05)70073-6

[4] Golding EM, Robertson CS, Bryan RM Jr (1999) The consequences of traumatic brain injury on cerebral blood flow and autoregulation: a review. Clin Exp Hypertens 21: 299–332. 10369378 10.3109/10641969909068668

[5] Werner C, Engelhard K (2007) Pathophysiology of traumatic brain injury. Br J Anaesth 99: 4–9. 17573392 10.1093/bja/aem131

[6] Coles JP, Fryer TD, Smielewski P, Chatfield DA, Steiner LA, et al (2004) Incidence and mechanisms of cerebral ischemia in early clinical head injury. J Cereb Blood Flow Metab 24: 202–211. 14747747 10.1097/01.WCB.0000103022.98348.24

[7] Cunningham AS, Salvador R, Coles JP, Chatfield DA, Bradley PG, et al (2005) Physiological thresholds for irreversible tissue damage in contusional regions following traumatic brain injury. Brain 128: 1931–1942. 15888537 10.1093/brain/awh536

[8] Diringer MN, Videen TO, Yundt K, Zazulia AR, Aiyagari V, et al (2002) Regional cerebrovascular and metabolic effects of hyperventilation after severe traumatic brain injury. J Neurosurg 96: 103–108. 11794590 10.3171/jns.2002.96.1.0103

[9] Stiefel MF, Tomita Y, Marmarou A (2005) Secondary ischemia impairing the restoration of ion homeostasis following traumatic brain injury. J Neurosurg 103: 707–714. 16266054 10.3171/jns.2005.103.4.0707

[10] Unterberg AW, Stover J, Kress B, Kiening KL (2004) Edema and brain trauma. Neuroscience 129: 1021–1029. 15561417 10.1016/j.neuroscience.2004.06.046

[11] Carbonell WS, Grady MS (1999) Evidence disputing the importance of excitotoxicity in hippocampal neuron death after experimental traumatic brain injury. Ann N Y Acad Sci 890: 287–298. 10668434 10.1111/j.1749-6632.1999.tb08005.x

[12] Robertson CL, Bell MJ, Kochanek PM, Adelson PD, Ruppel RA, et al (2001) Increased adenosine in cerebrospinal fluid after severe traumatic brain injury in infants and children: association with severity of injury and excitotoxicity. Crit Care Med 29: 2287–2293. 11801827 10.1097/00003246-200112000-00009

[13] Krajewska M, You Z, Rong J, Kress C, Huang X, et al (2011) Neuronal deletion of caspase 8 protects against brain injury in mouse models of controlled cortical impact and kainic acid-induced excitotoxicity. PLoS ONE 6: e24341. 21957448 10.1371/journal.pone.0024341 PMC3174961

[14] Clausen F, Marklund N, Lewen A, Enblad P, Basu S, et al (2012) Interstitial F(2)-isoprostane 8-iso-PGF(2alpha) as a biomarker of oxidative stress after severe human traumatic brain injury. J Neurotrauma 29: 766–775. 21639729 10.1089/neu.2011.1754

[15] Yamada KH, Kozlowski DA, Seidl SE, Lance S, Wieschhaus AJ, et al (2012) Targeted gene inactivation of calpain-1 suppresses cortical degeneration due to traumatic brain injury and neuronal apoptosis induced by oxidative stress. J Biol Chem 287: 13182–13193. 22367208 10.1074/jbc.M111.302612 PMC3339949

[16] Zhuang Z, Zhou ML, You WC, Zhu L, Ma CY, et al (2012) Hydrogen-rich saline alleviates early brain injury via reducing oxidative stress and brain edema following experimental subarachnoid hemorrhage in rabbits. BMC Neurosci 13: 47. 22587664 10.1186/1471-2202-13-47 PMC3436733

[17] Ramlackhansingh AF, Brooks DJ, Greenwood RJ, Bose SK, Turkheimer FE, et al (2011) Inflammation after trauma: microglial activation and traumatic brain injury. Ann Neurol 70: 374–383. 21710619 10.1002/ana.22455

[18] Acosta SA, Tajiri N, Shinozuka K, Ishikawa H, Grimmig B, et al (2013) Long-term upregulation of inflammation and suppression of cell proliferation in the brain of adult rats exposed to traumatic brain injury using the controlled cortical impact model. PLoS ONE 8: e53376. 23301065 10.1371/journal.pone.0053376 PMC3536766

[19] Johnson VE, Stewart JE, Begbie FD, Trojanowski JQ, Smith DH, et al (2013) Inflammation and white matter degeneration persist for years after a single traumatic brain injury. Brain 136: 28–42. 23365092 10.1093/brain/aws322 PMC3562078

[20] Tsai YD, Liliang PC, Cho CL, Chen JS, Lu K, et al (2013) Delayed neurovascular inflammation after mild traumatic brain injury in rats. Brain Inj 27: 361–365. 23438356 10.3109/02699052.2012.750738

[21] Clausen T, Khaldi A, Zauner A, Reinert M, Doppenberg E, et al (2005) Cerebral acid-base homeostasis after severe traumatic brain injury. J Neurosurg 103: 597–607. 16266040 10.3171/jns.2005.103.4.0597

[22] Gupta AK, Zygun DA, Johnston AJ, Steiner LA, Al-Rawi PG, et al (2004) Extracellular Brain pH and Outcome following Severe Traumatic Brain Injury. J Neurotrauma 21: 678–684. 15253796 10.1089/0897715041269722

[23] DeSalles AA, Kontos HA, Becker DP, Yang MS, Ward JD, et al (1986) Prognostic significance of ventricular CSF lactic acidosis in severe head injury. J Neurosurg 65: 615–624. 3772448 10.3171/jns.1986.65.5.0615

[24] Enevoldsen EM, Cold G, Jensen FT, Malmros R (1976) Dynamic changes in regional CBF, intraventricular pressure, CSF pH and lactate levels during the acute phase of head injury. J Neurosurg 44: 191–214. 1473 10.3171/jns.1976.44.2.0191

[25] Rabow L, DeSalles AF, Becker DP, Yang M, Kontos HA, et al (1986) CSF brain creatine kinase levels and lactic acidosis in severe head injury. J Neurosurg 65: 625–629. 3772449 10.3171/jns.1986.65.5.0625

[26] Vespa P, Bergsneider M, Hattori N, Wu HM, Huang SC, et al (2005) Metabolic crisis without brain ischemia is common after traumatic brain injury: a combined microdialysis and positron emission tomography study. J Cereb Blood Flow Metab 25: 763–774. 15716852 10.1038/sj.jcbfm.9600073 PMC4347944

[27] Wemmie JA, Price MP, Welsh MJ (2006) Acid-sensing ion channels: advances, questions and therapeutic opportunities. Trends Neurosci 29: 578–586. 16891000 10.1016/j.tins.2006.06.014

[28] Sherwood TW, Frey EN, Askwith CC (2012) Structure and Activity of the Acid Sensing Ion Channels. Am J Physiol Cell Physiol. 10.1152/ajpcell.00188.2012 PMC3469599 22843794

[29] Deval E, Gasull X, Noel J, Salinas M, Baron A, et al (2010) Acid-sensing ion channels (ASICs): pharmacology and implication in pain. Pharmacol Ther 128: 549–558. 20807551 10.1016/j.pharmthera.2010.08.006

[30] Xiong ZG, Pignataro G, Li M, Chang SY, Simon RP (2008) Acid-sensing ion channels (ASICs) as pharmacological targets for neurodegenerative diseases. Curr Opin Pharmacol 8: 25–32. 17945532 10.1016/j.coph.2007.09.001 PMC2267925

[31] Grunder S, Chen X (2010) Structure, function, and pharmacology of acid-sensing ion channels (ASICs): focus on ASIC1a. Int J Physiol Pathophysiol Pharmacol 2: 73–94. 21383888 PMC3047259

[32] Askwith CC, Wemmie JA, Price MP, Rokhlina T, Welsh MJ (2004) ASIC2 modulates ASIC1 H^+^-activated currents in hippocampal neurons. J Biol Chem 279: 18296–18305. 14960591 10.1074/jbc.M312145200

[33] Sherwood TW, Lee KG, Gormley MG, Askwith CC (2011) Heteromeric acid-sensing ion channels (ASICs) composed of ASIC2b and ASIC1a display novel channel properties and contribute to acidosis-induced neuronal death. J Neurosci 31: 9723–9734. 21715637 10.1523/JNEUROSCI.1665-11.2011 PMC3160670

[34] Weng JY, Lin YC, Lien CC (2010) Cell type-specific expression of acid-sensing ion channels in hippocampal interneurons. J Neurosci 30: 6548–6558. 20463218 10.1523/JNEUROSCI.0582-10.2010 PMC6632567

[35] Wemmie JA, Chen J, Askwith CC, Hruska-Hageman AM, Price MP, et al (2002) The acid-activated ion channel ASIC contributes to synaptic plasticity, learning, and memory. Neuron 34: 463–477. 11988176 10.1016/s0896-6273(02)00661-x

[36] Jasti J, Furukawa H, Gonzales EB, Gouaux E (2007) Structure of acid-sensing ion channel 1 at 1.9 A resolution and low pH. Nature 449: 316–323. 17882215 10.1038/nature06163

[37] Wemmie JA, Askwith CC, Lamani E, Cassell MD, Freeman JHJ, et al (2003) Acid-sensing ion channel 1 is localized in brain regions with high synaptic density and contributes to fear conditioning. J Neurosci 23: 5496–5502. 12843249 10.1523/JNEUROSCI.23-13-05496.2003 PMC6741257

[38] Yermolaieva O, Leonard AS, Schnizler MK, Abboud FM, Welsh MJ (2004) Extracellular acidosis increases neuronal cell calcium by activating acid-sensing ion channel 1a. Proc Natl Acad Sci U S A 101: 6752–6757. 15082829 10.1073/pnas.0308636100 PMC404117

[39] Xiong ZG, Zhu XM, Chu XP, Minami M, Hey J, et al (2004) Neuroprotection in ischemia: blocking calcium-permeable acid-sensing ion channels. Cell 118: 687–698. 15369669 10.1016/j.cell.2004.08.026

[40] Gu L, Liu X, Yang Y, Luo D, Zheng X (2010) ASICs aggravate acidosis-induced injuries during ischemic reperfusion. Neurosci Lett 479: 63–68. 20478356 10.1016/j.neulet.2010.05.029

[41] Friese MA, Craner MJ, Etzensperger R, Vergo S, Wemmie JA, et al (2007) Acid-sensing ion channel-1 contributes to axonal degeneration in autoimmune inflammation of the central nervous system. Nat Med 13: 1483–1489. 17994101 10.1038/nm1668

[42] Joch M, Ase AR, Chen CX, MacDonald PA, Kontogiannea M, et al (2007) Parkin-mediated monoubiquitination of the PDZ protein PICK1 regulates the activity of acid-sensing ion channels. Mol Biol Cell 18: 3105–3118. 17553932 10.1091/mbc.E05-11-1027 PMC1949385

[43] Pidoplichko VI, Dani JA (2006) Acid-sensitive ionic channels in midbrain dopamine neurons are sensitive to ammonium, which may contribute to hyperammonemia damage. Proc Natl Acad Sci U S A 103: 11376–11380. 16847263 10.1073/pnas.0600768103 PMC1544094

[44] Sun X, Cao YB, Hu LF, Yang YP, Li J, et al (2011) ASICs mediate the modulatory effect by paeoniflorin on alpha-synuclein autophagic degradation. Brain Res 1396: 77–87. 21529788 10.1016/j.brainres.2011.04.011

[45] Wong HK, Bauer PO, Kurosawa M, Goswami A, Washizu C, et al (2008) Blocking acid-sensing ion channel 1 alleviates Huntington’s disease pathology via an ubiquitin-proteasome system-dependent mechanism. Hum Mol Genet 17: 3223–3235. 18658163 10.1093/hmg/ddn218

[46] Zhao X, Gorin FA, Berman RF, Lyeth BG (2008) Differential hippocampal protection when blocking intracellular sodium and calcium entry during traumatic brain injury in rats. J Neurotrauma 25: 1195–1205. 18847376 10.1089/neu.2008.0635 PMC2652584

[47] Turner RJ, Van den Heuvel C, Vink R (2004) Amiloride increases neuronal damage after traumatic brain injury in rats. J Am Coll Nutr 23: 534S–537S. 15466959 10.1080/07315724.2004.10719397

[48] Carbonell WS, Maris DO, McCall T, Grady MS (1998) Adaptation of the fluid percussion injury model to the mouse. J Neurotrauma 15: 217–229. 9528921 10.1089/neu.1998.15.217

[49] Xiong Y, Mahmood A, Chopp M (2013) Animal models of traumatic brain injury. Nat Rev Neurosci 14: 128–142. 23329160 10.1038/nrn3407 PMC3951995

[50] Vink R, McIntosh TK, Yamakami I, Faden AI (1988) 31P NMR characterization of graded traumatic brain injury in rats. Magn Reson Med 6: 37–48. 3352504 10.1002/mrm.1910060105

[51] Ziemann AE, Allen JE, Dahdaleh NS, Dbrebot II, Coryell M, et al (2009) The amygdala is a chemosensor that detects hypercarbia and acidosis to elicit fear behavior. Cell 139: 1012–1021. 19945383 10.1016/j.cell.2009.10.029 PMC2808123

[52] Hallam TM, Floyd CL, Folkerts MM, Lee LL, Gong QZ, et al (2004) Comparison of behavioral deficits and acute neuronal degeneration in rat lateral fluid percussion and weight-drop brain injury models. J Neurotrauma 21: 521–539. 15165361 10.1089/089771504774129865

[53] Sato M, Chang E, Igarashi T, Noble LJ (2001) Neuronal injury and loss after traumatic brain injury: time course and regional variability. Brain Res 917: 45–54. 11602228 10.1016/s0006-8993(01)02905-5

[54] Sarkar S, Schmued L (2011) Fluoro-Jade dyes: fluorochromes for the histochemical localization of degenerative neurons. In: Bolon B, Butt MT, editors. Fundamental neuropathology for pathologists and toxicologists: principles and techniques. Hoboken: John Wiley & Sons. 171–179.

[55] Schmued LC, Albertson C, Slikker W Jr (1997) Fluoro-Jade: a novel fluorochrome for the sensitive and reliable histochemical localization of neuronal degeneration. Brain Res 751: 37–46. 9098566 10.1016/s0006-8993(96)01387-x

[56] Zhang B, Chen X, Lin Y, Tan T, Yang Z, et al (2011) Impairment of synaptic plasticity in hippocampus is exacerbated by methylprednisolone in a rat model of traumatic brain injury. Brain Res 1382: 165–172. 21276433 10.1016/j.brainres.2011.01.065

[57] Wu A, Molteni R, Ying Z, Gomez-Pinilla F (2003) A saturated-fat diet aggravates the outcome of traumatic brain injury on hippocampal plasticity and cognitive function by reducing brain-derived neurotrophic factor. Neuroscience 119: 365–375. 12770552 10.1016/s0306-4522(03)00154-4

[58] Harrison FE, Reiserer RS, Tomarken AJ, McDonald MP (2006) Spatial and nonspatial escape strategies in the Barnes maze. Learn Mem 13: 809–819. 17101874 10.1101/lm.334306 PMC1783636

[59] Lee DJ, Gurkoff GG, Izadi A, Berman RF, Ekstrom AD, et al (2013) Medial septal nucleus theta frequency deep brain stimulation improves spatial working memory after traumatic brain injury. J Neurotrauma 30: 131–139. 23016534 10.1089/neu.2012.2646

[60] Mouzon B, Chaytow H, Crynen G, Bachmeier C, Stewart J, et al (2012) Repetitive mild traumatic brain injury in a mouse model produces learning and memory deficits accompanied by histological changes. J Neurotrauma 29: 2761–2773. 22900595 10.1089/neu.2012.2498 PMC13588329

[61] Lehmann H, Rourke BK, Booker A, Glenn MJ (2012) Single session contextual fear conditioning remains dependent on the hippocampus despite an increase in the number of context-shock pairings during learning. Neurobiol Learn Mem. 10.1016/j.nlm.2012.10.011 23142771

[62] Koseki H, Matsumoto M, Togashi H, Miura Y, Fukushima K, et al (2009) Alteration of synaptic transmission in the hippocampal-mPFC pathway during extinction trials of context-dependent fear memory in juvenile rat stress models. Synapse 63: 805–813. 19504621 10.1002/syn.20657

[63] Curzon P, Rustay NR, Browman KE (2009) Cued and Contextual Fear Conditioning for Rodents. In: Buccafusco JJ, editor. Methods of Behavior Analysis in Neuroscience. 2nd ed. Boca Raton (FL). 21204331

[64] Szydlowska K, Tymianski M (2010) Calcium, ischemia and excitotoxicity. Cell Calcium 47: 122–129. 20167368 10.1016/j.ceca.2010.01.003

[65] Benson CJ, Xie J, Wemmie JA, Price MP, Henss JM, et al (2002) Heteromultimerics of DEG/ENaC subunits form H^+^-gated channels in mouse sensory neurons. Proc Natl Acad Sci U S A 99: 2338–2343. 11854527 10.1073/pnas.032678399 PMC122366

[66] Yagi J, Wenk HN, Naves LA, McCleskey EW (2006) Sustained currents through ASIC3 ion channels at the modest pH changes that occur during myocardial ischemia. Circ Res 99: 501–509. 16873722 10.1161/01.RES.0000238388.79295.4c

[67] Benson CJ, Sutherland SP (2001) Toward an understanding of the molecules that sense myocardial ischemia. Ann N Y Acad Sci 940: 96–109. 11458710 10.1111/j.1749-6632.2001.tb03669.x

[68] Baron A, Waldmann R, Lazdunski M (2002) ASIC-like, proton-activated currents in rat hippocampal neurons. J Physiol 539: 485–494. 11882680 10.1113/jphysiol.2001.014837 PMC2290154

[69] Ziemann AE, Schnizler MK, Albert GW, Severson MA, Howard MA (2008) Seizure termination by acidosis depends on ASIC1a. Nat Neurosci 11: 816–822. 18536711 10.1038/nn.2132 PMC2553357

[70] Nedergaard M, Kraig RP, Tanabe J, Pulsinelli WA (1991) Dynamics of interstitial and intracellular pH in evolving brain infarct. Am J Physiol 260: R581–588. 2001008 10.1152/ajpregu.1991.260.3.R581 PMC3062631

[71] Kraig RP, Ferreira-Filho CR, Nicholson C (1983) Alkaline and acid transients in cerebellar microenvironment. J Neurophysiol 49: 831–850. 6834101 10.1152/jn.1983.49.3.831

[72] Ishrat T, Sayeed I, Atif F, Hua F, Stein DG (2012) Progesterone is neuroprotective against ischemic brain injury through its effects on the phosphoinositide 3-kinase/protein kinase B signaling pathway. Neuroscience 210: 442–450. 22450229 10.1016/j.neuroscience.2012.03.008 PMC3358507

[73] Xiao G, Wei J, Yan W, Wang W, Lu Z (2008) Improved outcomes from the administration of progesterone for patients with acute severe traumatic brain injury: a randomized controlled trial. Crit Care 12: R61. 18447940 10.1186/cc6887 PMC2447617

[74] Bourdeaux CP, Brown JM (2011) Randomized controlled trial comparing the effect of 8.4% sodium bicarbonate and 5% sodium chloride on raised intracranial pressure after traumatic brain injury. Neurocrit Care 15: 42–45. 21298358 10.1007/s12028-011-9512-0

[75] Bourdeaux C, Brown J (2010) Sodium bicarbonate lowers intracranial pressure after traumatic brain injury. Neurocrit Care 13: 24–28. 20422466 10.1007/s12028-010-9368-8

[76] Muizelaar JP, Marmarou A, Ward JD, Kontos HA, Choi SC, et al (1991) Adverse effects of prolonged hyperventilation in patients with severe head injury: a randomized clinical trial. J Neurosurg 75: 731–739. 1919695 10.3171/jns.1991.75.5.0731

[77] Rosner MJ, Becker DP (1984) Experimental brain injury: successful therapy with the weak base, tromethamine. With an overview of CNS acidosis. J Neurosurg 60: 961–971. 6716165 10.3171/jns.1984.60.5.0961

[78] Wolf AL, Levi L, Marmarou A, Ward JD, Muizelaar PJ, et al (1993) Effect of THAM upon outcome in severe head injury: a randomized prospective clinical trial. J Neurosurg 78: 54–59. 8416243 10.3171/jns.1993.78.1.0054

[79] Yoshida K, Marmarou A (1991) Effects of tromethamine and hyperventilation on brain injury in the cat. J Neurosurg 74: 87–96. 1984513 10.3171/jns.1991.74.1.0087

[80] Marmarou A (1992) Intracellular acidosis in human and experimental brain injury. J Neurotrauma 9 Suppl 2S551–562. 1613813

